# Insulator Defect Detection via a Residual Denoising Diffusion Mechanism

**DOI:** 10.3390/ma18081738

**Published:** 2025-04-10

**Authors:** Li Zhang, Mengyang Song, Huaping Guo, Yange Sun, Xinxia Wang

**Affiliations:** 1 School of Computer Science and Artificial Intelligence, Zhengzhou University, Zhengzhou 450001, China; zhangli@xynu.edu.cn; 2 School of Computer and Information Technology, Xinyang Normal University, Xinyang 464000, China; songmengyang@xynu.edu.cn (M.S.); yangesun@xynu.edu.cn (Y.S.); wxx@xynu.edu.cn (X.W.); 3 Zhengzhou National Supercomputer Center, Zhengzhou 450001, China

**Keywords:** object detection, diffusion model, insulator defect, residual denoising diffusion

## Abstract

Insulators are critical components of transmission lines, and defective insulators pose a serious threat to the safety of power supply systems. Timely detection of these defects is crucial to prevent catastrophic consequences for human lives and property. However, insulator defects are often small and easily affected by the noise of rain, fog, sunlight, dirt, and other pollutants, making detection challenging. We observe that diffusion models learn data distribution by progressively introducing noise and subsequently performing denoising. The progressive denoising mechanism can naturally simulate the randomness of environmental noise. Based on this observation, we treat the localization of insulator defects as a denoising-based recovery process, where the true defect bounding boxes are progressively reconstructed from noisy representations. To this end, we propose a novel diffusion-based Insulator Defect Detector (IDDet) that is specifically designed to handle complex environmental noise. IDDet introduces noise to the true bounding boxes to generate noisy target boxes with random distributions and is then trained to recover the true bounding boxes from these noisy representations through a residual denoising diffusion mechanism. For the inference stage, IDDet refines the defect location from a random noise bounding box by gradually removing the noise, ultimately achieving the task of precisely locating the defect in the image. Experimental results show that IDDet significantly improves detection capability in noisy environments, achieving the best mean average precision (mAP) of 92.3%, confirming the feasibility and effectiveness of our approach.

## 1. Introduction

Insulators, key components of high-voltage transmission lines, play a crucial role in ensuring electrical insulation. However, harsh operating conditions and prolonged outdoor exposure may damage them, leading to issues like insulator detachment. Statistics indicate that over 75% of global power grid failures are attributed to insulators [1]. Therefore, regular inspection and maintenance are crucial for the stability of power systems.

Historically, detecting insulator defects relies heavily on manual inspection [2]. This process is time-consuming, labor-intensive, and prone to errors due to varying environmental conditions. Recently, deep-learning-based detectors have been successfully used for insulator defect detection by addressing noise interference from environmental factors like rain, fog, and dust [3]. For instance, Sadykova et al. [4] improved YOLOv2 with data augmentation to enhance the recognition of insulators obscured by elements such as ice and snow, while Zhang et al. [5] introduced SimAM attention into YOLOv5s to better extract key features from complex backgrounds. To further address noise interference from challenging weather conditions, adversarial training strategies have also been applied. Zhang et al. [6] implemented a generative adversarial network (GAN) to simulate adverse environmental effects such as rain and fog during training, enabling the model to become more resilient to real-world challenges.

**Figure 1:**
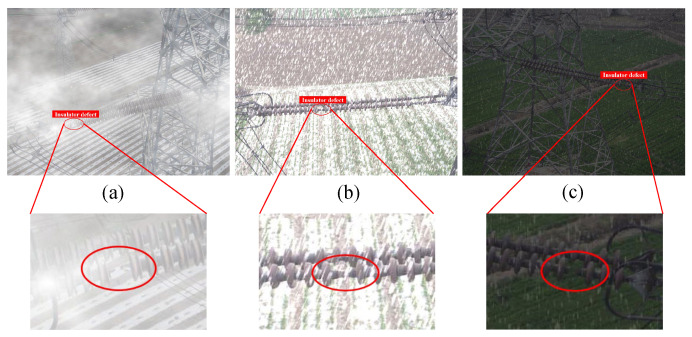
Samples of insulator defect images. Images are typically small and easily affected by environmental noise. (**a**) Fog. (**b**) Snow. (**c**) Sunlight and snow.

Despite the progress made by these methods in detecting insulator defects, challenges still remain in real-world environments due to various disruptive factors such as fog, rain, snow, intense sunlight, dirt, and pollutants, as illustrated in Figure 1. These environmental interferences not only hinder the clarity of captured images but also introduce significant noise, complicating the precise detection of insulator defects. Detecting small or subtle defects becomes particularly challenging in noisy outdoor settings where the contrast between the defect and its surroundings is reduced. Moreover, variations in lighting conditions, such as from the shadows and glare caused by sunlight, further exacerbate the problem by creating uneven illumination, which can obscure defect patterns. Similarly, atmospheric conditions like fog and heavy rain reduce image visibility, while pollutants and dirt accumulate on insulator surfaces, altering their appearance and making defect detection even more difficult. Snow and ice, on the other hand, can cover or partially obscure defects, resulting in missed detections. Therefore, the development of more precise and robust algorithms is imperative to effectively mitigate these environmental interferences and enhance the reliability of defect detection in real-world conditions.

Intuitively, removing noise from these images can restore the true distribution of these small insulator defects, thereby aiding in their precise identification. We observed that the diffusion model [7,8,9,10] is the correct method for generating images that align with the actual distribution through the denoising process. Thus, we treat defect localization as recovering bounding boxes from noisy images and introduce a diffusion model to detect insulator defects. However, existing diffusion models often struggle with small-object detection and highly noisy environments. To overcome this limitation, we introduce the Residual Denoising Diffusion Mechanism (RDDM) [11], which is specifically designed for insulator defect detection under challenging conditions. The RDDM dynamically adjusts the emphasis on target features during the denoising process, reducing the likelihood of small defects being obscured by background noise, while its independent noise diffusion path enhances the model’s robustness to non-Gaussian noise such as raindrops, fog, and glare. Based on the RDDM, we propose the Insulator Defect Detector (IDDet), an encoder–decoder network designed to detect defects in complex environmental noise. During training, noisy bounding boxes are created by adding Gaussian noise to true defect boxes, while in the prediction stage, the encoder extracts key features and the decoder iteratively refines the bounding box from an initial noisy state, filtering out noise boxes to precisely locate the defects.

In summary, the main contribution of the paper are summarized as follows:

- We propose an end-to-end encoder–decoder network, called IDDet, for insulator defect detection. Different from existing methods, IDDet formulates the detection task as a denoising process, where the encoder extracts key defect features from the input image and the decoder gradually recovers the true defect box from noisy bounding boxes.
- We introduce a Residual Denoising Diffusion Mechanism (RDDM) to dynamically emphasize target features during the denoising process, which not only reduces the chance of defects being masked by background interference but also improves robustness against complex non-Gaussian noise.
- Experimental results demonstrate that IDDet significantly improves detection performance in noisy environments, outperforming both traditional methods and state-of-the-art deep learning models.

The structure of the remainder of this paper is as follows: Section 2 reviews the related work, Section 3 details the proposed method, Section 4 presents the experimental results, Section 5 discusses the work in this paper, and, finally, the work is summarized in Section 6.

## 2. Related Work

### 2.1. Insulator Defect Detection

Currently, the existing object detection methods for identifying insulator defects can be broadly categorized into two types: single-stage and two-stage methods.

#### 2.1.1. Single-Stage Methods

Single-stage detection methods include the YOLO series [12,13,14,15,16] and SSD [17]. These methods predict bounding boxes and class probabilities directly from input images in a single forward pass, making them computationally efficient and well-suited for real-time applications.

For the detection of insulator defects, researchers have improved single-stage detection methods by introducing innovative convolutional structures, integrating efficient attention mechanisms, optimizing loss functions, and enhancing feature fusion. These enhancements significantly improve both the precision and efficiency of the detection process. For instance, Liu et al. [18] integrated YOLOv3 with CSPDarknet53, incorporating the CIOU loss function and the K-means++ clustering algorithm to improve the precision of detecting insulator defects. However, this improvement was achieved at the expense of a significant decrease in the detection speed. Bao et al. [19] adopted YOLOv5 as the base network architecture, enhanced the backbone network with a Channel Attention (CA) mechanism module, and integrated the architecture of the Bidirectional Feature Pyramid Network (Bi-FPN), thereby improving the precision of the insulator defect detection. Wang et al. [20] aimed to improve the detection precision of insulators by introducing Darknet53 to replace the original backbone network. However, they focused exclusively on the detection of insulators, overlooking the broader issue of detecting insulator defects. Han et al. [21] developed a D-CSPDarknet53 network to replace the YOLOv4 backbone, integrating the Shuffle Attention (SA) mechanism into the feature fusion network and introducing a new detection head to enhance insulator defect recognition. Huang et al. [22] achieved model lightweighting by pruning redundant layers from YOLOv5 and introduced an adaptive attention module between adjacent residual modules to enhance the network’s feature learning capability. Yi et al. [23] enhanced YOLOv5 by introducing GSConv, designing the VoV-GSCSP module and MaECA attention mechanism, optimizing the loss function in the Spatial Pyramid Pooling Module (SPPF), and incorporating the SIoU loss function.

#### 2.1.2. Two-Stage Methods

Two-stage detection algorithms [24,25,26] are primarily based on convolutional neural networks, generating a series of candidate boxes and then classifying and refining these candidate boxes. For the insulator detection issue, Shuang et al. [27] developed a detector based on Faster R-CNN, using a feature enhancement module to improve detail capture and an attention mechanism to refine target areas. Ou et al. [28] introduced a Faster R-CNN-based detection, enhancing the feature extraction network by removing some high-level convolutions from VGG16. Wang et al. [29] proposed a convolutional neural network-based railway insulator fault detection system that employs a cascade detection network and a fault classification network to identify faulty insulators in high-resolution, complex backgrounds.

Two-stage detection methods, while achieving more precise detection through candidate box generation and refinement, often suffer from slower inference speeds, making them less practical for real-time applications. Additionally, their robustness is limited in complex and noisy environments, where noise can obscure small targets and lead to decreased detection precision. These limitations highlight the trade-off between precision and efficiency in two-stage methods, emphasizing the need for further improvements to balance these aspects in practical scenarios.

### 2.2. Diffusion Model

A diffusion model is a latent variable model that uses a fixed Markov chain to map data to a latent space by progressively adding noise to reach a Gaussian equilibrium distribution. A Markov chain is a stochastic process in which the next state depends only on the current state and not on the sequence of states that preceded it. Mathematically, a Markov process satisfies the following property:

(1) $$P\left(z_{t}|z_{t-1},z_{t-2},\ldots ,z_{0}\right)=P\left(z_{t}|z_{t-1}\right)$$

where $z_{t}$ represents the state at time *t* and the transition probabilities depend only on the most recent state $z_{t-1}$. In real-world applications, noise is often composed of multiple independent sources, such as sensor noise, environmental interference, and quantization errors. According to the central limit theorem (CLT), when the effects of numerous independent random variables accumulate, their sum tends to follow a normal distribution, i.e., a Gaussian distribution [30]. This theoretical foundation justifies the assumption that noise in practical scenarios follows a Gaussian distribution, aligning with the diffusion model’s progressive noise addition process.

The training objective of the diffusion model is to learn the denoising process, enabling the generation of new data by traversing backward along the Markov chain. Let $z_{0}$ and $z_{T}$ be the original and noisy data at the final step of the Markov chain, respectively. The forward process of a diffusion model can be written as:

(2) $$z_{t}=\sqrt{1-\alpha_{t}}\epsilon_{t}+\sqrt{\alpha_{t}}z_{t-1},t=1,\ldots ,T$$

where $\alpha_{t}$ is the noise variance at each step and $\epsilon_{t}\;∼\;N\left(0,I\right)$ is a standard Gaussian random variable. The reverse process of a diffusion model can be written as:

(3) $$z_{t-1}=\frac{z_{t}-\sqrt{\alpha_{t}}\epsilon_{t}}{\sqrt{1-\alpha_{t}}},t=T,\ldots ,1$$

The denoising process aims to recover original data from noisy inputs and uses a neural network $p_{\theta }\left(z_{t-1},z_{t}\right)$ to predict the distribution of $z_{t-1}$ based on $z_{t}$, trained by maximizing the log-likelihood of the data.

(4) $$\log p_{\theta }\left(z_{0}\right)=\log p\left(z_{T}\right)+\sum_{t=1}^{T}\log p_{\theta }\left(z_{t-1}|z_{t}\right)$$

where $p(z_{T})$ is the prior distribution of the noisy data, usually assumed to be Gaussian.

Diffusion models have been successfully applied to many computer vision tasks. For example, Zhou et al. [31] introduced DiffDet4SAR for detecting aircraft in SAR images, effectively handling size variations. De et al. [32] proposed SEMI-DiffusionInst, using diffusion models to improve detection precision for semiconductor defects. Liu et al. [33] combined YOLO and diffusion models for noise-resistant object detection. They extracted feature maps from the denoising diffusion probability model to enhance the well-trained model, allowing the fine-tuning of YOLO on high-quality datasets and testing on low-quality datasets. Du et al. [34] proposed a diffusion model (ISTD-diff) for infrared small-target detection, iteratively generating target masks from noisy backgrounds. Pang et al. [35] introduced a decoupled diffusion network for detecting floating waste, incorporating a novel box update strategy to obtain the desired boxes during the inference stage. Zhang et al. [36] proposed an innovative crack detection framework, CrackDiff, based on a generative diffusion model. CrackDiff leverages the learning capabilities of a generative diffusion model to generate more precise and continuous crack segmentation results, focusing on the data distribution and latent spatial relationships of cracks across various sample time-steps.

In summary, diffusion models offer unique advantages in handling noise and small-object detection tasks by leveraging their progressive denoising mechanism. This capability makes them particularly effective in recovering true data distributions even under challenging, noisy conditions, providing a promising alternative for applications where conventional object detection methods struggle with precision and robustness.

## 3. Method

Insulators in working environments are often affected by factors like rain, fog, snow, and pollutants, generating noise that hinders detection. Their small size makes them more vulnerable to such interference. Effectively eliminating these noise disturbances is crucial for precise detection. We observe that diffusion models perform exceptionally well in tasks such as image denoising and restoration, effectively recovering even very small targets on insulators. The progressive denoising mechanism of diffusion models can naturally simulate the randomness of environmental noise. By introducing random Gaussian noise to perturb the ground truth boxes, the model learns to recover the target distribution under specific noisy scenarios. Therefore, we introduce the idea of using the diffusion model to aid in the detection of insulator defects and propose a novel insulator defect detector (IDDet) via a residual renoising diffusion mechanism.

### 3.1. Architecture

**Figure 2:**
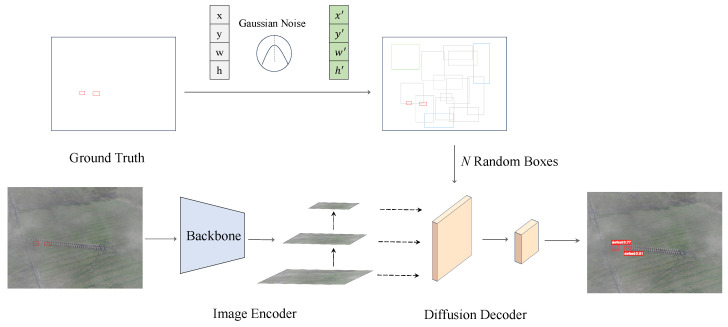
Framework of our proposed IDDet. The backbone extracts feature maps from the input insulator defects image. Taking noisy bounding boxes and multiscale features as input, the detector then predicts the target category, positions (center coordinates), and sizes (widths and heights) of the bounding boxes.

Figure 2 shows the overall architecture of our IDDet, which comprises three components: (1) a Noise Injection Module (NIM), (2) an image encoder, and (3) a diffusion decoder. The NIM introduces the idea of the Residual Denoising Diffusion Mechanism (RDDM) for noise bounding box generation. Specifically, the RDDM decouples diffusion into residual and noise diffusion, with independent coefficient schedules $\alpha_{t}$ controlling residual diffusion speed and $\beta_{t}^{2}$ regulating noise diffusion. In reverse, $\alpha_{t}$ and $\beta_{t}^{2}$ control the removal speed of residuals and noise, respectively. This approach helps eliminate noise interference and precisely locate insulator defects. The image encoder uses pre-trained backbone networks such as ResNet50 [37], ResNet101 [37], EfficientNet [38], Swin Transformer [39], and Pyramid Vision Transformer version 2 (PVTv2) [40] for feature extraction and integrates a Feature Pyramid Network (FPN) to enhance small target detection in insulators. The diffusion decoder, inspired by Sparse R-CNN [41], crops RoI features from the encoder’s feature map using proposal boxes and sends them to the detection head for target box regression and classification.

### 3.2. Image Encoder

The image encoder takes the raw image as input and extracts its high-level features for the following detection decoder, as shown in Figure 2 (bottom left). We implement the image encoder, which is compatible with a variety of advanced backbone networks, including but not limited to convolutional neural networks such as ResNet [37] and EfficientNet [38], as well as Transformer-based architectures like Swin Transformer [39] and Pyramid Vision Transformer version 2 (PVTv2) [40]. Here, we select ResNet50 as the backbone to capture features from insulator defect images at various levels, enabling a deep understanding of image content. Then, a feature pyramid network [42] is employed to enhance feature expression by integrating features from different levels.

### 3.3. Diffusion Decoder

**Figure 3:**
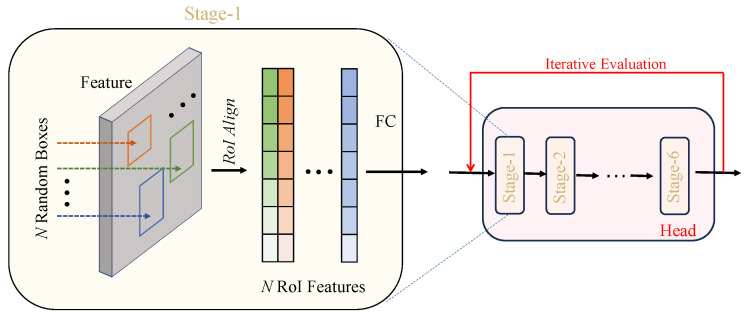
Details of the diffusion decoder.

Our diffusion decoder is composed of six cascading stages, as shown in Figure 3. Here, we use a decoder structure similar to that of Sparse R-CNN [43] to determine the target bounding boxes: the decoder takes a set of noisy boxes as the proposals and uses the proposals to crop the Region of Interest (RoI) features from the feature maps generated by the image encoder. Note: during the training stage, these noisy boxes are generated by adding Gaussian noise controlled by variance scheduling to the true boxes, whereas the noisy boxes are directly sampled from a Gaussian distribution during the inference stage. Then, these RoI features are sent to the detection decoder for predicting the true noise-free boxes from the noisy boxes. Our decoder differs from Sparse R-CNN in several ways: (1) starting inference with random boxes instead of fixed learned boxes, (2) requiring only proposal boxes during inference, without additional feature information, and (3) iteratively using the detection head with shared parameters, guided by the diffusion process through time-step embedding instead of employing the detection decoder only once during forward propagation. The denoising process in IDDet follows a Markov chain, where each noisy bounding box state $z_{t}$ depends only on the immediate previous state $z_{t-1}$, rather than on earlier states. This Markovian assumption ensures that the inference process remains efficient, as each denoising step only requires the previous state, reducing computational overhead. By iteratively refining bounding boxes in a structured manner, IDDet gradually removes noise while preserving defect-related features.

### 3.4. Noise Injection Module

In the noise injection module, we decouple the diffusion process into residual diffusion and noise diffusion. We use two independent coefficient schedules, $\alpha_{t}$ and $\beta_{t}^{2}$, to control the noise scale. In the early stages of the diffusion process, the coefficient $\alpha_{t}$ is set as a gradually decreasing positive sequence to ensure that the target features are progressively weakened but not completely lost. The noise intensity $\beta_{t}^{2}$ is independently set as a dynamically adjusted parameter of the Gaussian distribution, simulating a variety of complex environmental noises by increasing the noise variance. Thus, we define the single forward process step as follows:

(5) $$z_{t}=z_{t-1}+z_{res}^{t},\;z_{res}^{t}∼N\left(\alpha_{t}z_{res},\beta_{t}^{2}I\right)$$

where $z_{res}^{t}$ represents a directional mean shift (residual diffusion) with random perturbation (noise diffusion) from state $z_{t-1}$ to state $z_{t}$. Expanding Equation (5) yields formula Equation (6).

(6) $$\begin{array}{cc} z_{t} & =z_{t-1}+\alpha_{t}z_{res}+\beta_{t}\epsilon_{t-1}\\ \; & =z_{t-2}+\left(\alpha_{t-1}+\alpha_{t}\right)z_{res}+\sqrt{\beta_{t-1}^{2}+\beta_{t}^{2}}\epsilon_{t-2}\\ \; & =z_{0}+\bar{\alpha_{t}}z_{res}+\bar{\beta_{t}}\epsilon \end{array}$$

where ϵ ∼ N(0,I), ${\bar{\alpha }}_{t}=\sum_{i=1}^{t}\alpha_{i}$ and ${\bar{\beta }}_{t}=\sqrt{\sum_{i=1}^{t}\beta_{i}^{2}}$. The forward process can be defined as

(7) $$q\left(z_{t}|z_{0},z_{res}\right)=N\left(z_{t};z_{0}+{\bar{\alpha }}_{t}z_{res},{\bar{\beta }}_{t}^{2}I\right)$$

where $z_{res}$ represents the target residual features. Through the forward diffusion process, noise is added to the insulator defect images to generate noisy boxes.

### 3.5. Training

During training stage, we construct the diffusion process from ground-truth boxes to noisy boxes and train the model to reverse this process. In particular, we first pad the original ground-truth boxes with extra boxes to reach a fixed total of $N_{train}$. Then, the noise injection module introduces Gaussian noise to these padded boxes (see Section 3.4), generating $N_{train}$ noisy boxes. The diffusion decoder uses these noisy boxes to crop RoI features from the feature maps of the image encoder (see Section 3.2), which are then sent to the detection head (see Section 3.3) for box regression and classification. Finally, the predicted results are optimized through a set prediction loss function. This design ensures that the model’s training process is stable and consistent, effectively improving the model’s detection performance.

The insulator defect detection task involves input–target pairs (x,b,l), where *x* is the input image and *b* and *l* are the bounding box and class label, respectively. Each bounding box $b^{i}$ is represented by its center coordinates $\left(c_{x}^{i},c_{y}^{i}\right)$ and dimensions $\left(w^{i},h^{i}\right)$. From Equation (7), the data sample consists of *N* bounding boxes and $z_{0}=b\in R^{N\times 4}$, where each box is defined by its center coordinates, width, and height. Our IDDet encodes the input image *x* into feature maps $f_{\varphi }\left(x\right)$ and, using $z_{t}$ and $f_{\varphi }\left(x\right)$ as inputs, it decodes the true bounding box $z_{0}$ and class label *c* as $f_{\theta }\left(z_{t},f_{\varphi }\left(x\right)\right)$. The loss function is calculated using Equation (8)

(8) $$L_{train}=\frac{1}{2}{∥f_{\theta }\left(z_{t},f_{\varphi }\left(x\right)\right)-z_{0}∥}^{2}$$

where ${∥\cdot ∥}^{2}$ denotes the L2 norm (Euclidean distance).

Notably, the ground-truth box coordinates are scaled since the signal-to-noise ratio has a significant effect on the performance of diffusion model [44]. We experimentally observe that a scaling factor of 1.0 achieves the optimal mAP. There is more discussion of this in Section 4.5.3.

### 3.6. Inference

During the inference process, IDDet generates noisy boxes by sampling from a Gaussian distribution. The diffusion decoder takes noisy boxes and Region of Interest (RoI) features cropped from the feature map generated by the image encoder as input and refines the regression and classification results of the target box through iterative sampling, leveraging the denoising process learned during the training phase. With each step of the iterative process, the detection decoder is used to predict the current target bounding boxes, and the RDDM is employed to update these boxes for the subsequent prediction step. After each sampling step, the predicted boxes are categorized as either desired (properly located) or undesired (arbitrarily distributed). To maintain consistency with training, we filter out boxes with scores below a threshold and concatenate the remaining ones with new random boxes sampled from a Gaussian distribution. Thus, we can evaluate IDDet with an arbitrary number of random boxes and iteration times that do not need to be equal to the training stage.

## 4. Experiments

### 4.1. Experimental Setup

#### 4.1.1. Dataset

In this experiment, we utilize the publicly available insulator dataset proposed by Zhang et al., which is developed by Qilu University of Technology (Shandong Academy of Sciences), located in Jinan, Shandong, China [5]. They utilized the imgaug library on the AI Studio platform to simulate environmental conditions like rain, snow, fog, and low light, creating a new insulator dataset called WI that focuses on insulator shedding defects. To further validate the robustness of our results, we conducted a five-fold cross-validation experiment on our dataset. The dataset was randomly divided into five subsets, where four subsets were used for training and one for testing in each iteration. This process was repeated five times, and the final results were averaged to obtain stable performance metrics.

#### 4.1.2. Implement Details

**Figure 4:**
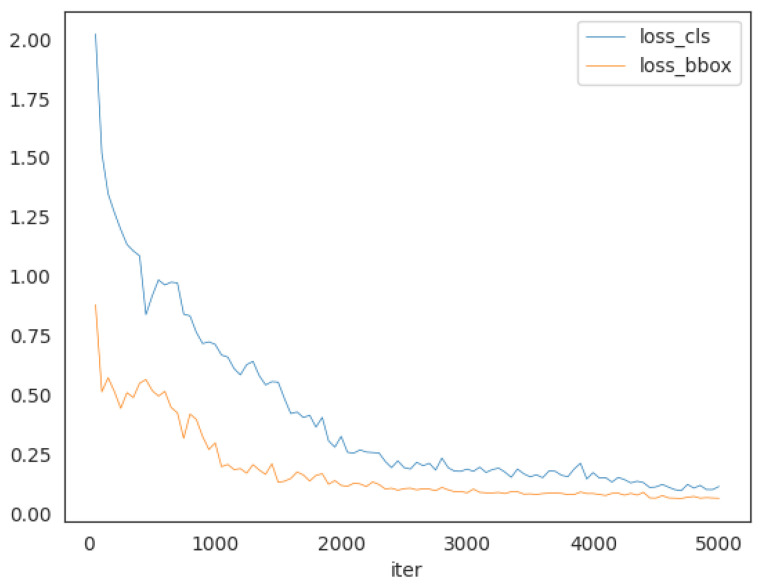
The classification loss and bounding box regression loss during training.

The experiments are conducted on a NVIDIA A100 GPU for both training and testing. The total number of parameters is approximately 25 million, requiring around 12 GB of GPU memory for training. Training IDDet on the WI dataset takes approximately 10 h using a single A100 GPU. Our IDDet is constructed using the PyTorch (version 1.13.0) framework and the Python (version 3.8.19) programming language. We use AdamW [45] as the optimizer, with an initial learning rate of 0.01. The optimizer’s momentum and weight decay values are configured to 0.937 and 0.0001, respectively. The batch size is set to 8, and all other parameters are set to default values. Our IDDet is trained for a maximum of 5000 iterations, with validation performed every 1000 iterations. As shown in Figure 4, both classification loss and bounding box loss decrease rapidly with the increasing number of iterations, ultimately stabilizing at a steady level by the end of training. Overall, the model exhibits good convergence, with significant reductions and stabilization in both loss functions.

#### 4.1.3. Evaluation Metrics

We evaluate our IDDet using metrics of recall (*R*), precision (*P*), mean average precision (mAP), F_1_-score, and frames per second (FPS). Recall (*R*) is the ratio of the number of samples correctly predicted as the positive class by the classifier to the total number of actual positive samples, defined as

(9) $$R=\frac{TP}{TP+FN}$$

where TP denotes instances where the model accurately predicts positive samples. FN represents cases where the model incorrectly predicts positive samples as negative.

Precision (*P*) is the ratio of the number of samples correctly predicted as the positive class by the classifier to the total number of samples classified as the positive class, defined as

(10) $$P=\frac{TP}{TP+FP}$$

where FP corresponds to situations where the model incorrectly predicts negative samples as positive.

The AP value measures the model’s performance within some specific category, defined as

(11) $$AP=\int_{0}^{1}P\left(R\right)dR$$

where *R* is the recall, measuring the model’s ability to detect positive samples, and *P* is the precision, evaluating the model’s predictions against actual outcomes.

The mAP is the mean AP, comprehensively evaluating the model’s performance by calculating the area enclosed by the precise recall curve for each class, defined as

(12) $$\mathrm{mAP}=\frac{1}{n}\sum_{i=0}^{n}AP(i)$$

The F_1_-score is the harmonic mean of precision and recall, which is used to evaluate the effectiveness of classification models, defined as

(13) $$F_{1}\text{-}\mathrm{score}=\frac{2\times P\times R}{P+R}$$

### 4.2. Backbone Network Effectiveness

To evaluate the effectiveness of different backbone networks for our proposed IDDet model, we conducted experiments using ResNet50 [37], ResNet101 [37], EfficientNet [38], Swin Transformer [39], and Pyramid Vision Transformer version 2 (PVTv2) [40]. We assess their performance in terms of mAP_50_, F_1_-score, and FPS, and the results are presented in Table 1.

As shown in Table 1, IDDet consistently achieves high performance across different backbone networks in terms of mAP_50_ and F1-score, demonstrating its robustness and insensitivity to the feature extraction capabilities of the backbone. With EfficientNet as the backbone, IDDet achieves the highest mAP50 (93.4%), while using Swin Transformer results in the best F_1_-score (92.3%). However, there are noticeable differences in FPS, which significantly affect real-time performance.

Among all the tested backbones, convolution-based networks such as ResNet50, ResNet101, and EfficientNet enable IDDet to achieve a better balance between precision and efficiency compared to Transformer-based architectures like Swin Transformer and PVTv2. Although Transformer-based models provide competitive precision, their inference speed is significantly slower, with Swin Transformer reaching only 40.9 FPS, making them less suitable for real-time applications.

Notably, with ResNet50 as the backbone, IDDet achieves the highest inference speed of 60.8 FPS while maintaining strong detection performance, with an mAP_50_ of 92.7% and an F_1_-score of 90.5%. This balance between speed and precision makes ResNet50 the most suitable choice for real-time defect detection tasks. Therefore, ResNet50 is selected as the backbone for IDDet to ensure both high precise and efficient inference.

### 4.3. Comparative Experiments

To comprehensively evaluate the effectiveness of our proposed IDDet, we compared it with nine state-of-the-art detection methods, including Faster RCNN [26], MobileNetv3-s [46], YOLOv4-tiny [14], YOLOv4 [14], YOLOv5s [47], YOLOv7 [16], TPH-YOLOv5s [48], SPD-Conv [49], and BS-YOLOv5s [5]. These methods represent a diverse range of detection paradigms, covering both single-stage and two-stage approaches, as well as anchor-based and anchor-free strategies. As shown in Table 2, IDDet outperforms all competing methods in terms of mAP_50_, F_1_-score, recall (R), and precision (P), demonstrating superior detection precision and robustness. Table 2 shows the 5-fold cross-validation results, where “•” indicates that IDDet significantly outperforms other methods, and “∘” denotes that IDDet is significantly outperformed by other methods, based on paired *t*-tests with a significance level of 0.05.

Compared to Faster R-CNN, a classic two-stage detection framework, IDDet achieves significantly better performance. Faster R-CNN achieves 82.8% in mAP_50_ and 64.5% in F1-score, with an inference speed of 14.5 FPS. In contrast, IDDet improves these metrics by 9.5% in mAP50 and 25.6% in F_1_-score, while increasing inference speed by 45.8 FPS. This highlights the limitations of two-stage methods in real-time defect detection, where IDDet offers a more precise and efficient alternative. When compared to BS-YOLOv5s, which achieves 89.8% in mAP_50_ and 88.9% in F1-score, IDDet further improves scores by 2.5% in mAP50 and 1.2% in F_1_-score. While BS-YOLOv5s has a slightly higher inference speed of 66.4 FPS compared to 60.3 FPS for IDDet, the superior detection precision of IDDet justifies the minor trade-off in speed, making it a more reliable choice for defect detection.

The advantages of IDDet are even clearer when compared to YOLOv5s, a widely used single-stage detector. YOLOv5s achieves 86.5% in mAP_50_, 88.1% in F1-score, and an inference speed of 57.6 FPS. IDDet surpasses these results, with improvements of 5.8% in mAP50, 2% in F_1_-score, and 2.7 FPS in speed. These findings show that IDDet combines high detection precision with real-time processing, which is crucial for practical applications. Although SPD-Conv achieves the highest inference speed of 81.9 FPS, its detection performance lags behind, with its mAP_50_ and F_1_-score reaching only 83.5% and 83.2%, respectively. Similarly, MobileNetv3-s has a high speed of 68.4 FPS but falls short in detection precision compared to IDDet.

To further evaluate the performance of IDDet, we considered precision (P) and recall (R) metrics. IDDet demonstrates remarkable improvements over other models, achieving 92.1% in precision and 80.6% in recall. This represents a significant increase compared to Faster R-CNN, which achieves 83.4% in precision and 76.0% in recall, with IDDet outperforming it by 8.7% in precision and 4.6% in recall. Compared to BS-YOLOv5s, which scores 91.4% in precision and 79.2% in recall, IDDet has improvements of 0.7% in precision and 1.4% in recall. YOLOv5s follows with 90.5% in precision and 73.2% in recall, with IDDet surpassing it by 1.6% in precision and 7.4% in recall.

In summary, these comparisons demonstrate that IDDet outperforms both traditional and modern detection methods, striking a strong balance between detection precision and inference speed. With its superior performance across all key metrics, IDDet proves to be a robust, efficient, and practical solution for insulator defect detection in challenging conditions such as rain, fog, and noisy backgrounds.

### 4.4. Visualization

**Figure 5:**
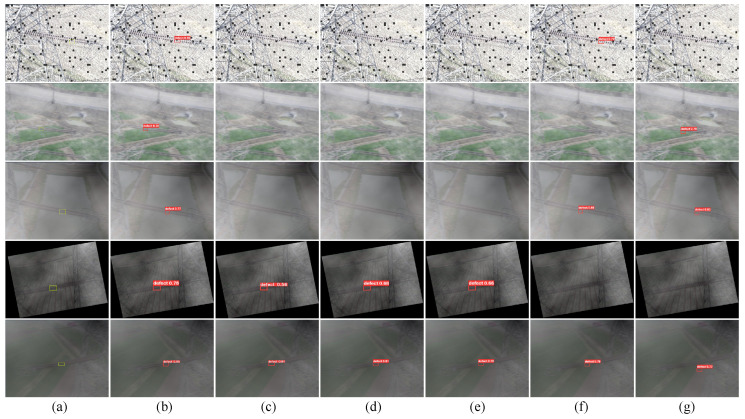
Visualization of detection resultsusing different methods on randomly selected images. Yellow boxes represent ground truths and red boxes indicate detection results. (**a**) Input images and ground-truth boxes, (**b**) IDDet, (**c**) BS-YOLOv5s, (**d**) YOLOv5s, (**e**) TPH-YOLOv5s, (**f**) SPD-Conv, and (**g**) Faster R-CNN.

We conducted a comprehensive visual evaluation of IDDet’s performance, as illustrated in Figure 5. The figure showcases the detection results using multiple methods, including IDDet, BS-YOLOv5s, YOLOv5s, TPH-YOLOv5s, SPD-Conv, and Faster R-CNN. Column (a) displays the original input images with yellow ground-truth bounding boxes. Columns (b)–(g) present the prediction results of each method, where red boxes indicate detected defects.

The images selected for visualization represent extremely challenging scenarios with various forms of visual degradation. The first row shows snow-covered insulator surfaces combined with strong glare, which makes defect edges difficult to distinguish. The second and third rows depict low-visibility environments caused by dense fog, where image contours are significantly blurred. The fourth row depicts a dimly lit scene with deep structural shadows, while the fifth row illustrates a hazy setting where a low signal-to-noise ratio significantly reduces defect visibility.

From Figure 5, IDDet demonstrates outstanding performance in detecting small insulator defects, highlighting its exceptional ability to accurately localize subtle targets even in the presence of severe visual degradation. This advantage is especially evident in the first three rows, where the defects are either heavily obscured or exhibit extremely low contrast due to factors such as snow, fog, or rain. In these extreme scenarios, IDDet successfully detects the defects, with confidence scores of 0.80, 0.70, and 0.77, respectively. In contrast, most baseline methods—including BS-YOLOv5s, YOLOv5s, and TPH-YOLOv5s—completely fail to identify any defects.

In rows 4 and 5, the environmental interference is relatively less severe, and most methods (such as BS-YOLOv5s, YOLOv5s, and TPH-YOLOv5s) are able to successfully detect the targets. However, SPD-Conv and Faster R-CNN still fail to identify the critical defect in row 4, possibly due to their limited adaptability to subtle scene variations. In row 5, although all methods produce detection results, only IDDet generates a bounding box that fully overlaps with the defect region. In contrast, the bounding boxes predicted by the other methods do not completely cover the defect area, indicating inferior localization precision.

### 4.5. Ablation Study

#### 4.5.1. Noise Separation in the RDDM

To evaluate the contribution of the noise separation strategy in the RDDM, we conduct an ablation study comparing two configurations: (1) using only $\alpha_{t}$-controlled residual diffusion without noise separation and (2) adopting the full RDDM strategy, including noise separation.

The experiments are conducted on the WI dataset, focusing on challenging scenarios with strong glare and complex backgrounds. As shown in Table 3, removing the noise separation mechanism led to a decrease in performance: mAP_50_ drops from 92.7% to 91.5%, F_1_-score decreases from 90.5% to 89.4%, and FPS declines from 60.8 to 58.8. These results demonstrate that the noise separation mechanism plays a crucial role in improving detection precision (mAP_50_ and F_1_-score) and inference speed (FPS), significantly enhancing the model’s robustness in high-noise and dynamic environments.

#### 4.5.2. Signal Scaling Factor in RDDM

In the Residual Denoising Diffusion Mechanism (RDDM), the signal scaling factor controls the signal-to-noise ratio (SNR) during the diffusion process and is crucial for the model’s sensitivity to signals and noise suppression. As shown in Table 4, the model IDDet achieves the optimal mAP value when the scaling factor is set to 1.0, as significant noise interference in the insulator defect image can obscure the target in a complex environment. On the other hand, an excessively low SNR, such as 0.1, leads to insufficient target information in the feature map, reducing detection performance. Therefore, adjusting the signal scaling factor is essential to accommodate different noise levels and target sizes.

#### 4.5.3. Matching Between $N_{train}$ and $N_{eval}$

$N_{train}$ and $N_{eval}$ (refer to Section 3.5) represent the number of random bounding boxes generated by the diffusion process during the training and evaluation phases, respectively. It should be noted that these boxes are noise-added bounding boxes derived from ground-truth annotations. Our IDDet has the appealing property of accepting an arbitrary number of random boxes.

To investigate the impact of the number of training boxes on the inference performance, we train our model with $N_{train}\in \left[100,\;300,\;500,\;1000\right]$ random noise boxes and then evaluated each of these models with $N_{eval}\in \left[100,\;300,\;500,\;1000\right]$, as summarized in Table 5. Regardless of the value of $N_{train}$, the precision remains steady. $N_{train}$ had a greater impact on the detection results than $N_{eval}$, and our IDDet tends to perform better with high values of $N_{train}$. This is because more training boxes allowed the model to learn additional texture and semantic features, enhancing detection. During validation, there are typically fewer targets in the insulator defect images than $N_{eval}$. As a result, many boxes without targets are suppressed and subsequently discarded, leading to a relatively small improvement in detection results as $N_{eval}$ increases.

## 5. Discussion

Detecting insulator defects in complex backgrounds remains a challenging task due to the difficulty in distinguishing fine-grained details when small defects are surrounded by significant noise or cluttered environments such as rain, snow, and fog. To address these problem, this paper introduces a diffusion-based framework, called IDDet, to enhance detection precision by leveraging the denoising capabilities of diffusion models. The success of IDDet may primarily be attributed to (1) the use of the Residual Denoising Diffusion Mechanism (RDDM) and (2) addition of random candidate prediction windows during the prediction phase.

The RDDM enhances defect detection by adopting a stepwise denoising approach, which reduces the complexity of transitioning directly from high-noise to low-noise conditions. This gradual refinement improves the stability and reliability of the denoising process, ensuring that small defects remain distinguishable even in noisy environments. By dynamically emphasizing target features and iteratively refining residual information, the RDDM suppresses noise more effectively, enabling precise defect localization under challenging conditions. In addition, the introduction of the residual mechanism in the RDDM significantly improves the quality of generated samples, particularly in maintaining robustness against noise. By leveraging residual information during iterative denoising, the RDDM enhances the preservation of subtle defect features while progressively reducing background noise. This mechanism ensures reliable performance in handling noise, enabling precise defect detection even in environments with complex, non-Gaussian disturbances such as raindrops, fog, or glare.

During the prediction phase, stochastically generated candidate boxes via Gaussian distribution may cover insulator defect areas. The probability of coverage increases proportionally with the number of sampling iterations. Consequently, defect detection becomes a fine-tuning process, i.e., candidate boxes are progressively adjusted based on Region of Interest (RoI) features cropped from the features generated by the image encoder. Therefore, an effective strategy to enhance IDDet’s performance is to increase the probability of overlap between the noisy boxes and defect areas. Table 5 validates the effectiveness of this strategy.

IDDet shows strong advantages in detecting insulator defects in complex backgrounds, but it has two main limitations. First, to increase the chances of candidate boxes covering defect areas, IDDet generates a large number of random candidate boxes, which greatly increases computational cost, especially for high-resolution images. Second, IDDet’s performance may lack stability. While IDDet is designed for noisy or cluttered environments like rain, snow, or fog, it struggles in scenarios where insulator targets are clear and the background is simple. In such cases, the use of random candidate boxes adds unnecessary computational effort and reduces the efficiency that standard detection methods could achieve.

Future work will explore integrating the two-stage conventional object detection paradigm with the stochastic candidate box strategy to balance efficiency and robustness. For scenarios where the targets are clear and the background is relatively simple, traditional object detection methods could be employed exclusively to reduce computational costs. Conversely, for challenging conditions involving noisy or cluttered environments, the stochastic candidate box mechanism could be dynamically activated during the prediction phase to enhance coverage probability. This adaptive detection strategy may optimize computational efficiency and ensure consistent performance across tasks of varying complexity, providing greater flexibility and scalability for real-world applications.

## 6. Conclusions

Insulator defects are small and highly susceptible to interference from rain, fog, sunlight, dust, and other pollutants; thus, the issue of detecting insulator defects remains a challenge task. This paper reviews the detection of insulator defects as the task of recovering and locating defects from numerous noisy environmental images and proposes a diffusion-based insulator defect detector called IDDet to address the localization of insulator defects in noisy environmental images. During training, IDDet diffuses the target bounding box from the ground truth to a random distribution, allowing the model to learn the reverse denoising process. In the inference phase, the model starts with randomly generated noisy boxes and progressively refines the defect locations for precise localization. Experimental results demonstrate that IDDet significantly outperforms mainstream detection models under noisy conditions, confirming the effectiveness and reliability of the proposed method.

## Figures and Tables

**Table 1:** Performance comparison of different backbone networks; the best result in each column is marked in boldface.

Model	$\mathrm{mA}P_{50}$	F_1_-Score	FPS
ResNet50 [37]	92.7	90.5	**60.8**
ResNet101 [37]	92.9	91.6	58.6
EfficientNet [38]	**93.4**	90.2	60.5
SwinTransformer [39]	90.1	**92.3**	40.9
PvtV2 [40]	90.6	90.5	50.5

**Table 2:** After five-fold cross-validation, the experimental results (mean ± std) of comparing methods in term of the mAP_50_, F_1_-score, FPS, precision (P), and recall (R). The best result in each column is marked in boldface.

Model	${\mathrm{mAP}}_{50}$	F_1_-Score	FPS	P	R
Faster R-CNN [26]	82.8 ± 0.8•	64.5 ± 1.0•	14.5 ± 1.2•	83.4 ± 0.4•	76.0 ± 1.2•
MobileNetv3-s [46]	75.0 ± 0.9•	76.1 ± 1.1•	68.4 ± 1.9•	79.7 ± 1.2•	74.2 ± 2.1•
YOLOv4-tiny [14]	76.3 ± 1.1•	68.2 ± 1.22•	41.1 ± 3.9•	81.6 ± 1.0•	75.1 ± 0.6
YOLOv4 [14]	71.5 ± 0.7•	51.6 ± 1.2•	17.9 ± 2.2•	77.0 ± 1.0•	70.6 ± 0.7•
YOLOv5s [47]	86.5 ± 1.2•	88.1 ± 2.5	57.6 ± 2.9•	90.5 ± 0.5•	73.2 ± 2.0•
YOLOv7 [16]	78.0 ± 0.7•	79.2 ± 1.5•	38.8 ± 3.1•	91.6 ± 1.2	70.2 ± 1.1•
TPH-YOLOv5s [48]	86.8 ± 1.3•	87.4 ± 1.3•	41.2 ± 2.1•	90.1 ± 0.7•	74.4 ± 1.3•
SPD-Conv [49]	83.5 ± 1.1•	83.2 ± 0.9•	**81.9 ± 2.8**•	88.9 ± 0.9•	77.3 ± 1.4•
BS-YOLOv5s [5]	89.8 ± 0.9•	88.9 ± 0.9	66.4 ± 2.6•	91.4 ± 0.8•	79.2 ± 1.0
IDDet(Ours)	**92.3 ± 1.2**	**90.1 ± 1.0**	60.3 ± 2.5	**92.1 ± 0.6**	**80.6 ± 1.1**

**Table 3:** Experimental results using RDDM. The best result in each column is marked in boldface.

Index	RDDM	${\mathrm{mAP}}_{50}$	F_1_-Score	FPS
(1)	No	91.5	89.4	58.8
(2)	Yes	**92.7**	**90.5**	**60.8**

**Table 4:** Signal scale. Normal scaling factor to improve detection performance (%). The best result in each column is marked in boldface.

Scale	mAP	${\mathrm{mAP}}_{50}$	${\mathrm{mAP}}_{75}$	${\mathrm{mAP}}_{s}$	${\mathrm{mAP}}_{m}$
0.1	44.1	88.1	30.1	15.1	41.9
1.0	**54.9**	**92.7**	**61.8**	**31.4**	**56.0**
2.0	46.2	90.6	41.2	12.7	46.9
3.0	46.0	89.5	40.5	12.6	45.5

**Table 5:** Matching between training and inference box numbers on insulator defect images (mAP/%).

	Eval	100	300	500	1000
Train					
100		53.9	53.7	53.7	53.7
300		53.6	54.0	53.6	53.6
500		54.9	54.9	54.9	54.9
1000		54.9	54.6	54.7	54.9

## Data Availability

Our code is available at https://github.com/hpguo1982/IDDet (accessed on 7 April 2025).
